# Dietary Nucleotides Enhance Neurogenesis, Cognitive Capacity, Muscle Function, and Body Composition in Older Adults: A Randomized, Triple-Blind, Controlled Clinical Trial

**DOI:** 10.3390/nu17091431

**Published:** 2025-04-24

**Authors:** Javier Gene-Morales, Alvaro Juesas, Angel Saez-Berlanga, Ezequiel G. Martin, Luis Garrigues-Pelufo, Brayan S. Sandoval-Camargo, Fernando Martin-Rivera, Iván Chulvi-Medrano, Pablo Jiménez-Martínez, Carlos Alix-Fages, Pedro Gargallo, Julio Fernandez-Garrido, Oscar Caballero, Agustín Jerez-Martínez, Juan C. Colado

**Affiliations:** 1 Department of Physical Education and Sports, University of Valencia, 46010 Valencia, Spain; javier.gene@uv.es (J.G.-M.); angel.saez@uv.es (A.S.-B.); fernando.martin-rivera@uv.es (F.M.-R.); ivan.chulvi@uv.es (I.C.-M.); juan.colado@uv.es (J.C.C.); 2 Research Group in Prevention and Health in Exercise and Sport (PHES), University of Valencia, 46010 Valencia, Spain; alvaro.juesastorres@uchceu.es (A.J.); ezequielgmnutricion@gmail.com (E.G.M.); luisgape@alumni.uv.es (L.G.-P.); brasanca@alumni.uv.es (B.S.S.-C.); p.jimenez@icen.es (P.J.-M.); c.alix@icen.es (C.A.-F.); pedro.gargallo@uv.es (P.G.); 3 Department of Education Sciences, CEU Cardenal Herrera University, 46115 Castellón, Spain; 4 ICEN Research Center, Department of Health Research, 38002 Santa Cruz de Tenerife, Spain; 5 Nursing Department, Faculty of Nursing and Podiatry, University of Valencia, 46010 Valencia, Spain; julio.fernandez@uv.es (J.F.-G.); oscar.caballero@uv.es (O.C.); 6 Faculty of Sports Sciences, Catholic University of Murcia (UCAM), 30107 Murcia, Spain

**Keywords:** gerontology, inflammatory modulation, redox balance, physical activity, neuroplasticity

## Abstract

Background/Objectives: this study evaluated the differential effects of two distinct dietary nucleotide supplements, combined with spontaneous physical activity, on neuromuscular, cognitive, and metabolic adaptations in older adults. Methods: Sixty-nine physically independent older adults (aged 60–75 years) were randomly assigned to three groups: (1) a yeast nucleotides formulation (YN) standardized in a high content of free nucleotides (>40%) rich in all macro and micro nutrients naturally occurring in yeast cell (amino acids, minerals and B-group vitamin); (2) a neuro-based formulation (NF) consisting of a blend of monophosphate nucleotides 5′; or (3) a placebo. Participants maintained their spontaneous physical activities without structured exercise during a 10-week intervention. Assessments included physical function, cognitive performance, body composition, quality of life, and serum biomarkers of oxidative stress, inflammation, and neurogenesis. Results: Both nucleotide-supplemented groups demonstrated significant improvements compared to placebo in physical performance (*p* ≤ 0.045), cognitive function (Trail Making Test B [TMT-B]: *p* ≤ 0.012), oxidative stress biomarkers (*p* ≤ 0.048), inflammatory cytokines (*p* ≤ 0.023), and quality-of-life parameters (*p* ≤ 0.047). Body composition remained stable in supplemented groups, whereas placebo increased fat mass (5.04%) and decreased muscle mass (−2.18%). Conclusions: Dietary nucleotide supplementation enhances the benefits of spontaneous physical activity across all measured variables in older adults, highlighting nucleotides as promising nutritional support for healthy aging. YN exhibited a trend toward greater inflammatory modulation, whereas NF showed a tendency toward enhanced neurotrophic effects and functional improvements, with a statistically significant improvement in the Timed Up and Go Test (*p* = 0.014). These findings underscore the potential for tailored nucleotide-based interventions to optimize distinct physiological domains in aging populations.

## 1. Introduction

Population aging has emerged as a critical global challenge, with individuals aged 60 years or older representing one of the fastest-growing demographic segments worldwide [1]. By 2050, older adults are projected to account for over 20% of the total population [1,2], a demographic transformation that raises pressing concerns regarding age-related declines in musculoskeletal health, cognitive capacity, and metabolic homeostasis [3,4,5]. Although structured exercise programs are well documented to ameliorate these age-related declines, primarily through enhancements in muscle strength, cardiovascular function, and neuromuscular coordination, many older adults do not engage in formal training regimens [6]. Instead, many engage in spontaneously active lifestyles, typically characterized by unstructured activities such as walking, household chores, or moderate recreational pursuits [7,8]. While these less-intensive activities often confer measurable health benefits, including improvements in mobility and cardiorespiratory metrics [9,10], additional strategies may be needed to counteract the progressive impairments that commonly accompany advancing age.

From a physiological standpoint, aging is accompanied by multiple challenges related to energy metabolism and oxidative balance. Accumulating oxidative damage can exacerbate fatigue, impair cellular function, and accelerate declines in both muscle performance and cognitive ability [11,12]. For example, markers of oxidative stress such as F_2_-isoprostanes provide insights into lipid peroxidation and cellular damage [13], whereas glutathione represents a critical endogenous antioxidant involved in cellular defense mechanisms against oxidative injury [14]. Additionally, aging is accompanied by a reduction in neurotrophic support, particularly reflected by decreased expression of brain-derived neurotrophic factor (BDNF), thereby exacerbating age-related cognitive decline [15,16]. BDNF serves as a key regulator of neuroplasticity by facilitating synaptic growth, maintenance, and remodeling processes vital for cognitive health [17]. Furthermore, chronic systemic inflammation, characterized by elevated levels of inflammatory mediators including interleukin-6 (IL-6) and tumor necrosis factor-alpha (TNF-α), along with mitochondrial dysfunction associated with increased reactive oxygen species (ROS), further compromises skeletal muscle integrity, neuronal function, and metabolic regulation [18]. Therefore, nutritional interventions aimed at bolstering antioxidant defenses and enhancing recovery mechanisms are essential for mitigating the adverse effects associated with aging [19,20,21].

Within this context, nucleotide supplementation has emerged as a promising adjunct strategy. Nucleotides are low molecular weight compounds consisting of a nitrogenous base, a pentose sugar, and one or more phosphate groups, playing essential roles in cellular energy metabolism, immune modulation, and tissue repair [22]. Although nucleotides are endogenously synthesized, certain tissues exhibit limited de novo synthesis capacity, rendering them reliant on exogenous sources during periods of increased physiological demand [23,24]. Indeed, both preclinical and clinical studies have demonstrated that exogenous nucleotides enhance intestinal integrity, modulate inflammatory responses, and support immune cell activity [22,25,26,27,28,29]. Rodent models further suggest that nucleotides exert potent antioxidant and anti-aging properties, potentially mitigating deoxyribonucleic acid (DNA) damage, reducing memory deterioration, and preserving cellular homeostasis by improving mitochondrial function and reducing oxidative stress [30,31,32,33].

Research in human or clinical populations suggests that nucleotide supplementation may provide significant ergogenic and immunomodulatory benefits. In healthy adults, supplementation have been associated with improved exercise performance, enhanced recovery, and reduced systemic inflammation, likely due to increased energy utilization and immune function modulations [34,35,36,37]. In clinical populations, exogenous nucleotide administration has been shown to promote gut mucosal integrity, foster beneficial microbiota, and support immune resilience in conditions such as inflammatory bowel disease and infection-related immune suppression [25,27]. While these findings highlight the potential of nucleotide supplementation in maintaining physiological homeostasis, differences in nucleotide formulations could influence their specific physiological effects. Variations in nucleotide composition, such as the inclusion of the different ribonucleotide types or additional bioactive compounds, may impact bioavailability, mitochondrial function, and cellular signaling pathways involved in muscle repair and immune modulation [22]. Given the potential mechanistic distinctions between formulations, further research is warranted to compare their efficacy in modulating neuromuscular resilience, cognitive function, and metabolic adaptations in aging individuals with unstructured physical activity patterns.

To address these research gaps, this study aimed to (I) evaluate the effects of a spontaneously active lifestyle, with and without dietary nucleotide supplementation, on the functional, cognitive, and metabolic profiles of older adults; and (II) compare the efficacy of two distinct nucleotide formulations: a yeast nucleotides formulation (YN), that combines nucleotides with additional bioactive compounds (e.g., amino acids, B-group vitamins) and a neuro-based formulation (NF) consisting exclusively of monophosphate nucleotides, both tested against a placebo. The study also examined whether changes in inflammatory and oxidative stress biomarkers correlated with improvements in physical performance and cognitive function. We hypothesized that older adults receiving nucleotide supplementation would exhibit more pronounced enhancements in functional capacity, cognitive performance, and metabolic resilience compared to those in the placebo group. Specifically, YN was expected to be more effective in modulating inflammation and maintaining redox balance, while NF was anticipated to provide distinct neurotrophic benefits that further support cognitive function. The findings of this study may contribute to identifying effective nutritional strategies to promote healthy aging and offer novel insights for clinicians and exercise professionals seeking to implement feasible interventions in physically independent older populations.

## 2. Materials and Methods

### 2.1. Participants

**Figure 1:**
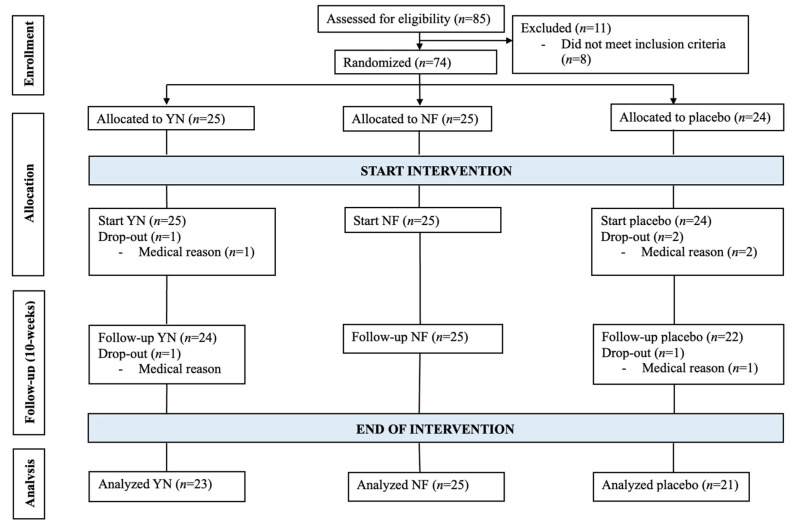
Participants flowchart. YN: yeast nucleotides. NF: neuro-based formulation.

An a priori analysis using G*Power (Version 3.1.9.3) determined that a sample size of 66 participants was required for a two-way ANOVA with three groups, ensuring 95% statistical power, an effect size of 0.25, and accounting for a 5% type I error. To account for potential sample dispersion within this population, a total of 74 participants of both sexes were recruited, of whom 69 completed the study (age: 67.99 ± 4.73 years; height: 167.51 ± 5.35 cm; weight: 73.11 ± 10.62 kg; body mass index: 26.07 ± 4.00 kg/m^2^; body fat percentage: 36.10 ± 6.71%; weekly physical activity: 719.92 MET × min/week). Participants included in this study were classified as sedentary who maintained an active lifestyle within their community, but did not engage in systematic physical conditioning exercise. Additionally, baseline conditions were strictly monitored to ensure consistency, with no differences between groups over the weeks. All dropouts were due to unrelated medical reasons, and not associated with injuries or adverse events linked to the intervention (see Figure 1 for details). Participants were randomly assigned to one of three groups: a YN group (n = 23), a NF group (n = 25), or a placebo group (n = 21). To ensure researcher blinding, external staff assigned an alphanumeric code to each participant and supplement group. Participants were unaware of the protocols followed by other groups or whether they received nucleotides supplementation or placebo.

Participants were eligible for inclusion in the study if they met the following criteria: (i) sedentary adults aged 60 or older who maintained an active lifestyle within their community, but do not engage in systematic physical conditioning exercise; (ii) being capable of signing an informed consent form; (iii) had refrained from taking antioxidant supplements (e.g., vitamins C, E, A, omega-3, etc.) for at least six weeks before the study began; and (iv) regular consumption of a balanced diet. Exclusion criteria included: (i) severe cardiopulmonary disease; (ii) neuromuscular or musculoskeletal disorders that hindered maintaining an active lifestyle within the community; (iii) a history of malignant neoplasms or a terminal illness with a life expectancy of less than a year; (iv) endocrine or metabolic disorders affecting muscle mass or bone mineral density; (v) use of medications within the last six months or currently that could influence bone mineral density and redox status (e.g., hormone replacement therapy, calcitonin, corticosteroids, glucocorticoids, allopurinol); (vi) smoking more than five cigarettes per day; (vii) a score below 23 on the Mini-Mental State Examination; or (viii) participation in another study involving dietary, exercise, or pharmaceutical interventions within the last six months. All potential participants were informed of the study’s purpose, procedures, benefits, risks, and potential discomforts before enrollment. Informed consent was obtained from each participant, who retained the right to withdraw from the study at any time. The study adhered to the Declaration of Helsinki, received approval from the University of Valencia Ethics Committee (IRB: 1861154), and was registered at ClinicalTrials.gov (NCT06620666).

### 2.2. Study Design

**Figure 2:**
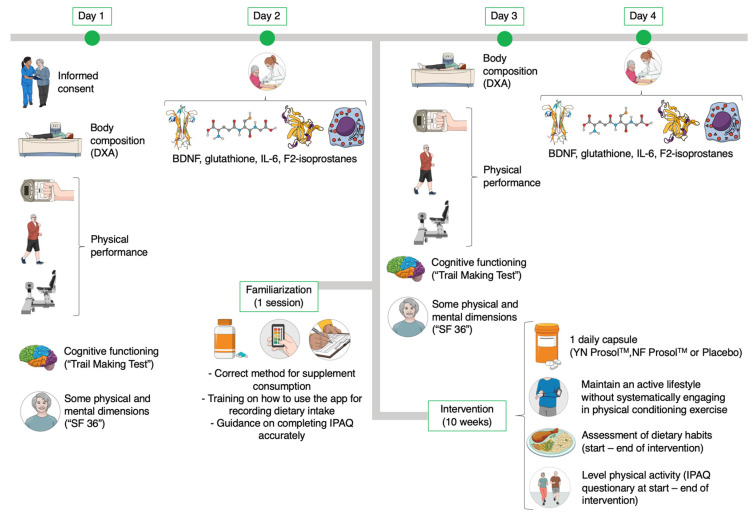
Graphical summary of the study procedures. Created with Mindthegraph.com. DXA: dual-energy X-ray absorptiometry. SF-36: 36-Item Short Form Health Survey. BDNF: brain-derived neurotrophic factor. IPAQ: International Physical Activity Questionnaire. YN: yeast nucleotides. NF: neuro-based formulation.

A triple-blind longitudinal study was designed to explore the effects of two nucleotide supplementation protocols (YN vs. NF) on functional capacities, isokinetic leg and arm strength, neurogenesis and oxidative stress parameters, inflammatory markers, body composition, cognitive status, and quality of life in older adults. All three groups (YN, NF, and placebo) completed (i) an initial testing session, (ii) a familiarization session, (iii) a 10-week intervention, and (iv) a final testing session. During the 10-week intervention, participants maintained an active lifestyle within their community, but did not engage in systematic physical conditioning exercise while consuming the randomly assigned product (supplement or placebo). Both initial and final testing included assessments of functional capacities, isokinetic leg and arm strength, neurogenesis and oxidative stress parameters, inflammatory markers, body composition, cognitive status, and quality of life. The study design is presented in Figure 2. All testing sessions were conducted at approximately the same time of day (within a 1 to 2 h window) under consistent environmental conditions.

### 2.3. Testing Procedures

#### 2.3.1. General Testing Protocol

Both initial and final testing sessions were conducted over two days at the University of Valencia by blinded staff who were not involved in the intervention. Participants were instructed to fast for eight hours, refrain from caffeine consumption, and avoid intensive exercise for 24 h before testing. Initial testing took place before the familiarization session, while final testing was carried out within 48 h after completing the 10-week intervention. On the first testing day, participants visited the Performance Laboratory at the Faculty of Physical Activity and Health Sciences between 8:30 and 10:00 a.m. Upon arrival, they remained seated for five minutes to standardize baseline conditions.

#### 2.3.2. Anthropometric and Body Composition Assessment

Height was measured to the nearest 0.1 cm using a portable stadiometer (Seca GmgH & Co. KG, Hamburg, Germany), and body weight using an electronical bascule (Seca 878 model; Seca GmbH & Co. KG, Hamburg, Germany). Body fat percentage and muscle mass were assessed using fan beam dual-energy X-ray absorptiometry (DXA) (QDR^®^ Hologic Discovery Wi, Hologic Inc., Waltham, MA, USA) with APEX software (version 12.4, Waltham, MA, USA). Participants wore light clothing and removed any metallic objects to ensure accuracy.

#### 2.3.3. Physical Performance Assessment

The characteristics of the tests performed on the first day of initial and final testing were as follows.

Physical performance was assessed using five tests: (i) maximal grip strength, following the protocol suggested by Roberts et al. [38]; (ii) isokinetic strength of knee and elbow flexors and extensors in the dominant leg and arm, measured with a multi-joint isokinetic dynamometer (Biodex Medical™, Shirley, NY, USA) using Advantage software (version 3.2, Shirley, NY, USA). The test was conducted within a range of motion from 5° to 90° at an angular velocity of 60°/s, with participants performing five consecutive flexion and extension movements; (iii) 30 s Chair Stand Test, evaluating local muscular endurance; (iv) 6 min Walk Test, assessing aerobic capacity; and (v) Timed Up and Go Test, measuring proactive balance and agility. The last three tests were selected from the Senior Fitness Test Battery [39], where they are thoroughly described.

#### 2.3.4. Cognitive Function Assessment

Cognitive assessment was conducted using the Trail Making Test (TMT) A and B, a neuropsychological test designed to evaluate executive functioning, processing speed, mental flexibility, scanning, and visual search [40]. In TMT-A, participants were instructed to connect numbers sequentially in ascending order (1 → 2 → 3, etc.), whereas in TMT-B, they alternated between numbers and letters in ascending order (1 → A → 2 → B, etc.). Completion time served as the primary outcome measure for both test conditions.

#### 2.3.5. Health-Related Quality of Life Assessment

Health-related quality of life assessment was performed using four key dimensions of the 36-Item Short Form Health Survey (SF-36), specifically general health, social functioning, vitality, bodily pain (i.e., primarily age-related musculoskeletal discomfort associated with daily movement), and mental health, to assess participants’ health status. Each dimension was evaluated by coding, aggregating, and transforming responses into a scale from 0 to 100, where 0 represents the poorest health status and 100 represents the best. Since the SF-36 is not designed to produce an overall score, a separate score was generated for each dimension [41]. Previous research has demonstrated the reliability and effectiveness of the SF-36 in assessing health-related quality of life in older adults [42].

#### 2.3.6. Blood Sample Collection and Biochemical Analysis

For blood sample collection, participants visited the Faculty of Nursing, where trained nurses performed the procedure. Blood samples were collected using serum-separating tubes (SSTs) (Greiner Bio-One GmbH, Kremsmünster, Austria), which facilitate the separation of serum from blood cells during centrifugation. Samples were centrifuged at 3000 rpm for 15 min at 4 °C, and the resulting serum was stored at −80 °C until further analysis. The concentrations of 8-isoprostane PGF_2_α (a type of F_2_-isoprostanes), BDNF, glutathione (i.e., GPx1), IL-6, and TNF-α were determined using enzyme-linked immunosorbent assays (ELISA). The kits used for these analyses were EIA-3080 (average intra-assay variability < 11.00%, inter-assay variability < 11.00%), EIA-5968 (average intra-assay variability < 9.00%, inter-assay variability < 8.50%), IL E-3200 (average intra-assay variability < 5.50%, inter-assay variability < 8.50%), IL E-3100 (average intra-assay variability < 5.00%, inter-assay variability < 8.00%), and EK241437 (average intra-assay variability < 5.00%, inter-assay variability < 7.50%), from DRG Instruments GmbH (Frauenbergstr, Marburg, Germany) (F_2_-isoprostanes and BDNF), LDN Labor Diagnostika Nord GmbH & Co. (Nordhorn, Germany) (IL-6 and TNF-α), and AFG Scientific (Northbrook, Illinois, USA) (GPx1). All laboratory procedures followed the Clinical and Laboratory Standards Institute (CLSI) guidelines.

#### 2.3.7. Familiarization Session

Following the initial testing, participants completed a familiarization session to ensure proper adherence to the study protocol. This session provided instructions on the correct method for supplement consumption, training on how to use the designated app for recording dietary intake, and guidance on completing the International Physical Activity Questionnaire (IPAQ) accurately.

### 2.4. Procedures for Monitoring Supplementation, Diet, and Physical Activity

Participants were randomly assigned to receive either one daily capsule of nucleotides or a placebo. Beginning on the first day of the intervention, participants consumed one capsule with their meal at 2:00 p.m. The YN supplement (RiboDiet^®^; Prosol S.r.l., Madone, Italy) contained, as the sole active ingredient, 250 mg of yeast extract obtained through a standardized and highly controlled process without organic solvents. It is a source of at least 40% nucleotides, including adenosine monophosphate (5′AMP), cytidine monophosphate (5′CMP), uridine monophosphate (5′UMP), and guanosine monophosphate (5′GMP), along with nucleosides, oligonucleotides, ribonucleic acid fragments, amino acids, minerals, and B-group vitamins. Meanwhile, the NF supplement (Ribocare^®^ Neuro; Prosol S.r.l., Madone, Italy) contained as the sole active ingredient 150 mg of four monophosphate nucleotides: 5′AMP, 5′CMP, 5′UMP, and 5′GMP. Both supplements are registered trademarks at the European Union level. The total daily dosage followed recommendations from the Spanish Agency for Food Safety and Nutrition, which advises a maximum intake of 450 mg of this type of supplement [43]. This dosage also aligns with previous human studies that have analyzed nucleotide supplementation and reported no adverse health effects [37,44,45]. The placebo capsules, identical in appearance to the nucleotide supplements, only contained maltodextrin and cellulose as excipient. To prevent participants from distinguishing the supplements based on sensory characteristics, the capsules were prepared under controlled conditions. Furthermore, participants, researchers, and statisticians remained blinded to the supplementation condition throughout the study. The technical staff responsible for capsule preparation stored them in independent facilities (INDIEX, Madrid, Spain) until analysis to maintain blinding.

For dietary control, all participants were instructed to maintain their usual dietary habits throughout the study. Dietary intake was assessed at both baseline and post-intervention over three non-consecutive days (two weekdays and one weekend day) using the validated smartphone app MyFitnessPal (MyFitnessPal, LLC, San Francisco, CA, USA) [46,47]. An experienced nutritionist supervised the use of the app and the accuracy of dietary recording to ensure compliance and reliability.

Physical activity and sedentary behavior in older adults can be assessed using subjective self-report methods or objective device-based approaches [48]. While self-reports, such as questionnaires, are cost-effective and easy to administer, they may be influenced by recall bias and social desirability [48]. Given the variations in activity type and intensity, subjective methods can provide sufficient detail while remaining practical and cost-efficient [48]. However, large-scale studies require valid and reliable tools to ensure consistency across different populations. The IPAQ is a widely used self-report instrument designed to estimate physical activity and sedentary behavior in adults aged 15 to 69 across diverse socioeconomic settings [49]. In this study, physical activity levels were assessed before and after the intervention using the validated Spanish version of the short-form IPAQ [50]. For each category of self-reported physical activity, energy expenditure was estimated in metabolic equivalents (METs) [51]. This estimation included total MET expenditure from walking, moderate-intensity activities (e.g., carrying light weights, cycling), vigorous-intensity activities (e.g., swimming, jogging, lifting heavy objects, padel) expressed in METs × min/week, and sedentary behavior expressed in min/day [50]. According to the IPAQ scoring protocol, physical activity was classified into three levels: low, moderate, or high [52]. Specifically, total physical activity was categorized as low (<600 METs × min/week), moderate (600–3000 METs × min/week), or high (>3000 METs × min/week, or at least 1500 METs × min/week of moderate activity combined with vigorous activity on a minimum of three days per week) [53,54]. Only activities lasting at least 10 min were considered [51].

### 2.5. Statistical Analysis

Descriptive data are presented as means and standard deviations (SDs). The Shapiro–Wilk test revealed that the 6 min Walk test, fat mass, and TNF-α were the only variables normally distributed. Percent difference (%Δ = [(post-test score − pre-test score)/pretest score] × 100) was calculated to assess changes in dependent variables between pre-test and post-test. A two-way repeated measures analysis of variance (ANOVA) was conducted, with time as a within-subject factor (pre-test vs. post-test) and group as a between-subject factor (YN vs. NF vs. Placebo) for normally distributed variables that were equal across groups at baseline (i.e., 6 min Walk Test, fat mass, and TNF-α). Subsequently, a two-way analysis of covariance (ANCOVA) was employed to normally distributed variables that were not equal between groups at the beginning of the study. This adjustment was made by including baseline values of the 6 min Walk Test and fat mass as covariates to control for potential initial differences affecting intervention outcomes [55,56].

In cases where a significant F-ratio was obtained, 95% confidence intervals were applied to examine differences between the adjusted ANCOVA pre- and post-test scores within groups, as well as post-test differences between groups. The Least Significant Difference (LSD) test was employed for post hoc comparisons, with effect sizes (ES) evaluated using partial eta squared (ƞp^2^).

The Kruskal–Wallis test revealed significant differences at both pre-test and post-test for all variables that deviated from normal distribution. These variables included the 30 s Chair Stand Test, Timed Up and Go Test, maximal grip strength, isokinetic leg and arm flexion-extension at 60°/s, TMT-A and TMT-B, body fat percentage, body muscle mass, vitality, general health, mental health, bodily pain, weekly METs × min (total, vigorous, moderate, and walking), sedentary time per day, total calories, protein, carbohydrates, lipids, glutathione, BDNF, F_2_-isoprostanes, and IL-6. The magnitude of these differences was assessed using ƞp^2^. Quade’s rank analysis of covariance (Quade’s ANCOVA) was then performed using baseline values of maximal grip strength, isokinetic leg and arm flexion-extension at 60°/s, TMT-B, body muscle mass, and BDNF as covariates to adjust for pre-existing differences, thereby enhancing the precision of comparisons across groups [57,58].

When the statistical significance was reached, 95% confidence intervals were used to determine meaningful differences between the adjusted Quade’s ANCOVA means at pre- and post-intervention stages, as well as post-intervention differences between groups. The Friedman Test assessed the effects of time as a within-participant factor, with ES evaluated using Kendall’s Coefficient of Concordance (*w*) following Cohen’s guidelines [59]. Post hoc comparisons were conducted using Wilcoxon tests for within-group analyses and Mann–Whitney U tests for between-group analyses, with ES calculated using Hedge’s g.

Bivariate correlation analysis was conducted to explore relationships between changes (Δ) in the dependent variables following the intervention. Since some variables did not meet normality assumptions, Spearman’s rank-order correlation was employed. Changes in variables were calculated as the difference between post- and pre-intervention values. The analysis focused on determining whether improvements or deteriorations in one variable were associated with changes in others, providing insights into potential interdependencies. Missing data in this study were addressed using multiple imputation, following the intention-to-treat method, in which baseline measurements for participants who withdrew were carried forward to the post-intervention phase [60]. The qualitative interpretation of ƞp^2^ was as follows: values between 0.01 and 0.06 indicated a small effect, values from 0.06 to 0.14 represented a medium effect, and values greater than 0.14 corresponded to a large effect. ES was calculated using Hedge’s *g* to account for potential small sample bias and was interpreted according to Cohen’s guidelines: trivial effect (<0.20), small effect (0.20–0.50), moderate effect (0.50–0.80), and large effect (>0.80) [59]. All statistical analyses were performed using SPSS software (IBM SPSS version 28.0.1.1(14), Armonk, NY, USA). The alpha level was set at a *p* < 0.05.

## 3. Results

### 3.1. Group Characterstics

Out of the 69 participants who completed the study, 65 completed the IPAQ. The assessment of the METs × min/week allowed for the classification of participants into two groups based on physical activity levels: moderate physical activity (808.07 ± 143.28 METs × min/week, n = 45) and sedentary behavior (356.66 ± 178.73 METs × min/week, n = 20). It is important to note that the classification for moderate physical activity ranges from 600 to 3000 METs × min/week. Therefore, despite 45 participants being classified as moderately active, the group’s average result was close to the sedentary threshold (<600 METs × min/week). Additionally, since none of the participants reached the minimum METs × min/week required for high physical activity levels, this indicates that the entire participant group exhibits low levels of weekly physical activity.

The analysis of the three study groups revealed no significant differences over time in any of the IPAQ variables, including vigorous METs × min/week (Friedman *p* = 0.739, YN group n = 3, NF group n = 4, placebo group n = 2), moderate METs × min/week (Friedman *p* = 0.483, YN group n = 15, NF group n = 16, placebo group n = 12), walking METs × min/week (Friedman *p* = 0.294, YN group n = 20, NF group n = 22, placebo group n = 18), total METs × min/week (Friedman *p* = 0.385, YN group = 661.73 ± 61.35 METs × min/week, NF group = 783.40 ± 31.49 METs × min/week, placebo group = 715.71 ± 42.46 METs × min/week), and sedentary time per day (Friedman *p* = 0.132, YN group = 256.52 min/day, NF group = 252.80 min/day, placebo group = 274.28 min/day). Likewise, there were no between-group differences throughout the study (Kruskal–Wallis *p* range = 0.304–0.866).

Self-reported adherence to the supplementation protocol was 90.77%. No significant differences were observed at baseline in age, weight, height, body mass index (BMI), or dietary intake (protein, carbohydrates, and lipids) between groups (*p* > 0.05 for all variables). Similarly, there were no significant differences in total caloric intake or macronutrient consumption between groups throughout the study (*p* range: 0.394–0.985).

### 3.2. Neurogenesis and Oxidative Stress Parameters

A significant time × group interaction was observed for BDNF (*F* = 5.71, *p* = 0.050, ηp^2^ = 0.20), indicating different trends between the experimental and placebo groups. Specifically, BDNF levels increased in the NF group compared to the placebo group. A significant main effect of time was also detected for BDNF (χ^2^ = 10.57, *p* = 0.001, *w* = 0.18). Pre-post comparisons showed significant improvements in BDNF levels in the experimental groups, while the placebo group did not exhibit a significant change. Additionally, a significant time × group interaction was found for F_2_-isoprostanes (H = 6.01, *p* = 0.041, ηp^2^ = 0.48), and glutathione (H = 5.92, *p* = 0.048, ηp^2^ = 0.48), indicating higher concentrations in both experimental groups compared to the placebo. A significant main effect of time was observed for F_2_-isoprostanes (χ^2^ = 50.45, *p* < 0.001, *w* = 0.73) and glutathione (χ^2^ = 29.35, *p* < 0.001, *w* = 0.43), with both experimental groups showing significant changes. Detailed pairwise comparisons are presented in Table 1.

### 3.3. Inflammatory Parameters

A significant time × group interaction was observed for IL-6 (H = 11.11, *p* = 0.004, ηp^2^ = 0.26), and TNF-α (*F* = 22.85, *p* < 0.001, ηp^2^ = 0.27), with both experimental groups exhibiting significantly lower levels compared to the placebo group. Specifically, the NF group showed significant reductions in IL-6, with a moderate effect size (ES = −0.52) compared to the placebo. A significant main effect of time was also found for IL-6 (χ^2^ = 7.67, *p* = 0.006, *w* = 0.13), and TNF-α (*F* = 10.25, *p* < 0.001, ηp^2^ = 0.24). Both inflammatory markers significantly decreased in the experimental groups, while the placebo group showed no significant changes, maintaining stable concentrations throughout the study. Pairwise comparisons are detailed in Table 2.

### 3.4. Functional Capacities

A significant time × group interaction was observed for all physical performance parameters, indicating greater improvements in the experimental groups compared to the placebo group, whose performance either remained stable or slightly declined. The significant interactions were as follows: 30 s Chair Stand Test (H = 15.13, *p* < 0.001, ηp^2^ = 0.36), Timed Up and Go Test (H = 19.42, *p* < 0.001, ηp^2^ = 0.25), 6 min Walk Test (*F* = 546.94, *p* < 0.001, ηp^2^ = 0.89), maximal grip strength (H = 8.24, *p* = 0.016, ηp^2^ = 0.16). A significant main effect of time was also found for most physical performance parameters, reflecting overall improvement from baseline to the end of the 10-week intervention: 30 s Chair Stand Test (χ^2^ = 3.95, *p* = 0.047, *w* = 0.06), Timed Up and Go Test (χ^2^ = 4.19, *p* = 0.041, *w* = 0.06), and 6 min Walk Test (*F* = 8.51, *p* = 0.005, ηp^2^ = 0.13). The only exception was maximal grip strength, which did not show a significant main effect of time (χ^2^ = 1.51, *p* = 0.218, *w* = 0.02). The Timed Up and Go Test exhibited a significant between-group effect, with NF showing the greatest improvement compared to YN (*p* = 0.014, ES = 0.79), and placebo (*p* < 0.001, ES = −1.50). Improvement percentages and effect sizes were notably higher in the NF group compared to the other groups (median ES = 0.54 for NF, 0.28 for YN, and 0.10 for the placebo group). Detailed pairwise comparisons are presented in Table 3.

### 3.5. Isokinetic Leg and Arm Strength

The time × group interaction was significant for all isokinetic strength variables, including leg flexion strength (H = 7.47, *p* = 0.024, ηp^2^ = 0.46), leg extension strength (H = 5.84, *p* = 0.043, ηp^2^ = 0.20), arm flexion strength (H = 5.93, *p* = 0.041, ηp^2^ = 0.20), and arm extension strength (H = 7.72, *p* = 0.021, ηp^2^ = 0.20). A significant effect of time was also observed for all these variables, with leg flexion strength (χ^2^ = 41.92, *p* < 0.001, *w* = 0.62), leg extension strength (χ^2^ = 49.47, *p* < 0.001, *w* = 0.73), arm flexion strength (χ^2^ = 24.00, *p* < 0.001, *w* = 0.35), and arm extension strength (χ^2^ = 62.06, *p* < 0.001, *w* = 0.91) all demonstrating significant improvements across the intervention. Although no significant differences were found between the experimental groups, effect sizes and percentage improvements in knee and arm flexion were consistently higher in the supplemented groups compared to the placebo group. Specifically, knee flexion increased by 13.77% and 11.29% in the YN and NF groups, respectively, compared to 6.27% in the placebo group. Similarly, elbow flexion strength increased by 4.29% and 13.11% in the experimental groups, while the placebo group experienced only a 2.16% improvement. In contrast, significant differences were observed for knee and elbow extension strength in favor of the nucleotide-supplemented groups. Knee extension strength increased by 0.60 and 0.56 ES in the YN and NF groups, respectively, compared to only 0.19 in the placebo group. Similarly, elbow extension strength improved with ES values of 0.54 and 0.60 in the supplemented groups, whereas the placebo group showed only a 0.19 ES improvement. Detailed pairwise comparisons are presented in Table 4.

### 3.6. Body Composition

The time × group interaction was significant both for fat mass (*F* = 5720.81, *p* < 0.001, ηp^2^ = 0.99) and for body fat percentage (H = 19.03, *p* < 0.001, ηp^2^ = 0.72), except for muscle mass (H = 4.17, *p* = 0.124, ηp^2^ = 0.72), indicating significant improvements in fat mass and body fat percentage in experimental groups compared to the placebo group. A significant effect of time was found for fat mass (*F* = 111.21, *p* < 0.001, ηp^2^ = 0.77), muscle mass (χ^2^ = 17.75, *p* < 0.001, *w* = 0.26), and body fat percentage (χ^2^ = 9.29, *p* = 0.002, *w* = 0.15). While both nucleotide-supplemented groups maintained stable fat mass and muscle mass levels throughout the intervention, the placebo group experienced a significant increase in fat mass along with a concurrent decline in muscle mass over time. Pairwise comparisons revealed that the most pronounced fat loss occurred in the NF group, but no significant differences were observed in muscle mass gains between the groups. Detailed pairwise comparisons are presented in Table 5.

### 3.7. Cognitive Functioning

A significant time × group interaction was observed for TMT-B (H = 10.32, *p* = 0.006, ηp^2^ = 0.51), but not for significant differences were found for TMT-A (H = 1.07, *p* = 0.587, ηp^2^ = 0.39). The improvements observed in TMT-B indicated superior executive functioning, processing speed, mental flexibility, visual scanning, and search abilities in the nucleotide-supplemented groups compared to the placebo group. Additionally, a significant main effect of time was found for both TMT-A (χ^2^ = 36.71, *p* < 0.001, *w* = 0.53) and TMT-B (χ^2^ = 31.12, *p* < 0.001, *w* = 0.45), demonstrating overall cognitive improvements from baseline to the 10-week follow-up. Detailed pairwise comparisons are presented in Table 6.

### 3.8. Physical and Mental Health-Related Quality of Life Dimensions

The time × group interaction was significant across all assessed dimension of the SF-36: general health (H = 10.00, *p* = 0.007, ηp^2^ = 0.45), bodily pain (H = 15.21, *p* < 0.001, ηp^2^ = 0.37), vitality (H = 8.21, *p* = 0.016, ηp^2^ = 0.32), and mental health (H = 11.44, *p* = 0.003, ηp^2^ = 0.21). A significant effect of time was also found for all dimensions, with general health (χ^2^ = 41.14, *p* < 0.001, *w* = 0.60), bodily pain (χ^2^ = 44.00, *p* < 0.001, *w* = 0.64), vitality (χ^2^ = 53.07, *p* < 0.001, *w* = 0.77), and mental health (χ^2^ = 55.25, *p* < 0.001, *w* = 0.80) all showing significant improvements. Improvement percentages and effect sizes were significantly greater in the experimental groups compared to the placebo group, with a median ES of 1.32 and 1.50 for YN and NF, respectively, versus 0.60 for the placebo group. Pairwise comparisons are presented in Table 7.

### 3.9. Bivariate Correlation Analysis (Spearman’s ρ)

**Figure 3:**
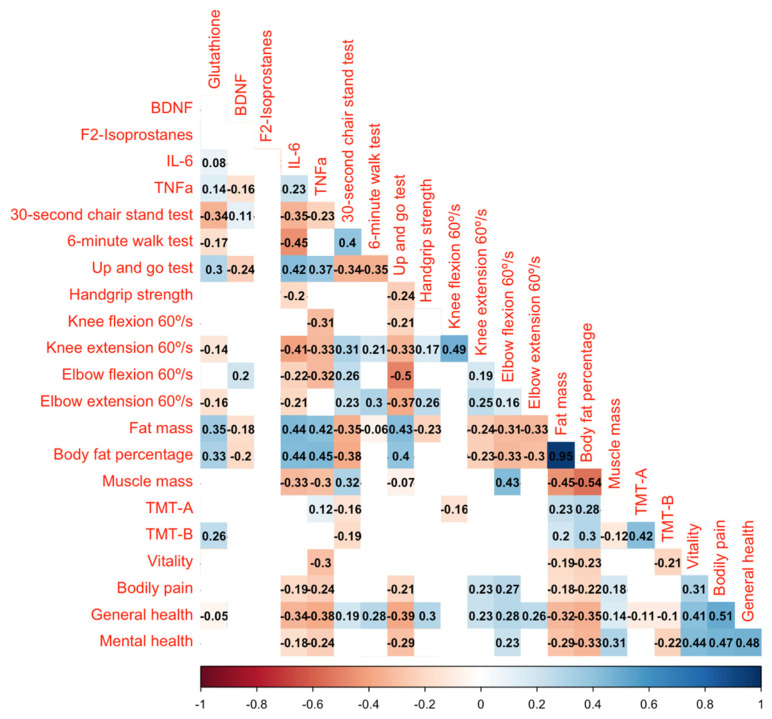
Correlation between changes in dependent variables pre-intervention and post-intervention. Only statistically significant correlations are represented. The magnitude of the correlation is depicted by the color intensity, ranging from negative to positive correlation (X-axis), along with the Spearmans’s *ρ*.

The correlation analysis revealed key associations linking inflammatory profile, oxidative stress, body composition, and physical performance parameters. Specifically, glutathione, BDNF, F_2_-isoprostanes, IL-6, and TNF-α exhibited significant relationships, highlighting their roles in physiological adaptations (see Figure 3 for more details).

Regarding inflammation and oxidative stress profiles, glutathione was positively correlated with BDNF (*ρ* = 0.08), reinforcing its potential neuroprotective and cellular resilience functions. Conversely, glutathione showed negative correlations with F_2_-isoprostanes (*ρ* = −0.16), IL-6 (*ρ* = −0.35), and TNF-α (*ρ* = 0.08), suggesting its role in reducing oxidative stress and systemic inflammation. Furthermore, a strong correlation between IL-6 and TNF-α (*ρ* = 0.40) highlights their interconnected role as pro-inflammatory cytokines.

Respecting body composition and physical performance, fat mass and body fat percentage were positively correlated with IL-6 (*ρ* = 0.42 and *ρ* = 0.45, respectively), indicating that greater adiposity may contribute to chronic inflammation. Conversely, muscle mass was negatively correlated with IL-6 (*ρ* = −0.33) and positively correlated with knee extension strength at 60°/s (*ρ* = 0.49), emphasizing the importance of muscle preservation in mitigating inflammation and enhancing physical function. Handgrip strength was negatively correlated with TNF-α (*ρ* = −0.24), IL-6 (*ρ* = −0.37), and F_2_-isoprostanes (*ρ* = −0.30), demonstrating the detrimental effects of oxidative stress and inflammation on neuromuscular performance. Stronger correlations were observed between knee extension at 60°/s and functional tests, including the 6 min Walk Test (*ρ* = 0.40) and the Timed Up and Go Test (*ρ* = −0.35), Reinforcing the role of lower-limb strength in mobility and functional independence. Additionally, BDNF was positively correlated with muscle mass (*ρ* = 0.26), further supporting its role in neuromuscular adaptations.

Concerning cognitive function, TMT-A demonstrated a significant negative correlation with the 6 min walk test (*ρ* = −0.35) and knee extension at 60°/s (*ρ* = −0.33), suggesting that greater lower limb function is linked to enhanced cognitive processing speed. Similarly, TMT-B showed negative associations with muscle mass (*ρ* = −0.30) and knee flexion at 60°/s (*ρ* = −0.31), indicating that higher neuromuscular performance corresponds with improved cognitive flexibility and executive function. In terms of perceived health, vitality was positively correlated with handgrip strength (*ρ* = 0.37), knee flexion at 60°/s (*ρ* = 0.42), and muscle mass (*ρ* = 0.33), highlighting the role of physical capacity in sustaining energy levels. Conversely, bodily pain exhibited negative correlation with knee extension at 60°/s (*ρ* = −0.35) and the 6 min walk test (*ρ* = −0.30), suggesting that reduce musculoskeletal function may contribute to increased pain perception. Additionally, general health showed significant positive correlations with knee extension strength (*ρ* = 0.41), handgrip strength (*ρ* = 0.38), and the 6 min walk test (*ρ* = 0.44), reinforcing the role of musculoskeletal fitness in overall health perception. Lastly, mental health demonstrated strong positive associations with vitality (*ρ* = 0.44) and general health (*ρ* = 0.47), indicating that improvements in subjective well-being are closely linked to both physical and psychological factors.

These findings highlight the complex interplay between oxidative stress, inflammation, body composition, and physical function, cognitive function and subjective well-being emphasizing the importance of maintaining muscle mass and redox balance for overall health in aging populations.

## 4. Discussion

The present study aimed to evaluate the effects of dietary nucleotide supplementation, in conjunction with a spontaneously active lifestyle, on enhancing neurogenesis, cognitive function, physical performance, body composition, and oxidative stress regulation in physically independent older adults. Additionally, the study sought to compare the efficacy of YN and NF, assessing their potential differences in modulating neuromuscular resilience, cognitive function, and metabolic adaptations.

The main findings of this study revealed that nucleotide supplementation significantly improved key markers of neurogenesis, oxidative balance, and overall functional capacity. Specifically, experimental groups exhibited a marked increase in BDNF levels, suggesting enhanced neurogenesis and cognitive resilience, along with favorable shifts in oxidative stress parameters, with reductions in F_2_-isoprostanes and increases in glutathione levels. Additionally, inflammatory markers such as IL-6 and TNF-α were significantly reduced in the nucleotide-supplemented groups compared to placebo, indicating a robust anti-inflammatory response. Furthermore, the experimental groups demonstrated significant improvements in physical performance, as evidenced by enhanced outcomes in the 30 s Chair Stand, Timed Up and Go, 6 min Walk Test, and isokinetic strength assessments. These functional gains were accompanied by favorable changes in body composition, reflected by maintenance in fat mass and body fat percentage, while cognitive performance, particularly in executive function as assessed by the TMT-B, also improved. Correlation analyses further emphasized the integrative role of enhanced redox balance and inflammatory modulation in both neuromuscular performance and cognitive function.

**Figure 4:**
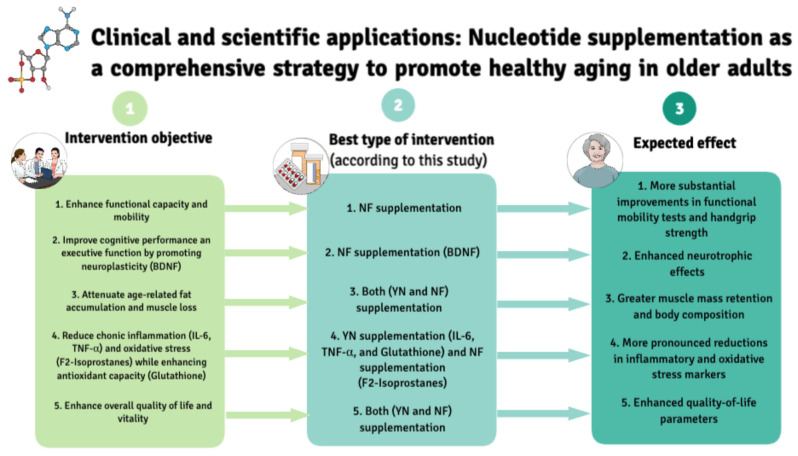
Key points of study. This table provides a concise summary of the main expected effects of this supplementation in older adults with an active lifestyle, based on percentage changes in each variable according to statistical significance and effect size. For a more comprehensive statistical interpretation of this figure, the reader is referred to the tables provided in the Results section. YN: yeast nucleotide formulation. NF: neuro-based formulation.

Overall, these findings suggest that dietary nucleotide supplementation, when combined with spontaneous physical activity, can effectively support neuromuscular and cognitive health, reinforcing its potential as a promising adjunct strategy for promoting healthy aging in older adults. While no significant differences were observed between the two supplementations groups, distinct trends emerged in their effects. NF supplementation exhibited a tendency toward greater neurotrophic benefits, as evidenced by the more pronounced increase in BDNF levels and improved performance in the TMT-B. Conversely, YN supplementation showed a stronger trend in enhancing neuromuscular recovery and oxidative stress modulation, with higher effect sizes in isokinetic strength measures and a slightly greater reduction in F_2_-isoprostanes. These findings suggest that although both formulations provide beneficial effects, their mechanisms of action may differ, and further research is warranted to determine whether longer intervention periods or different supplementation strategies could optimize their respective benefits in aging populations. Beyond clinical and scientific implications, these findings may also inform healthcare policymaking. As aging demographics continue to rise, cost-effective and scalable strategies that promote functional independence and cognitive health are increasingly relevant. Nutritional interventions such as nucleotide supplementation, particularly when combined with spontaneous physical activity, could be incorporated into preventive health frameworks or public aging programs aimed at reducing long-term healthcare burden and supporting healthy longevity. Figure 4 highlights the main findings and key takeaways of this study.

### 4.1. Spontaneous Activity and Supplementation in Older Adults

Previous research investigating the impact of spontaneous physical activity in older adults has demonstrated modest improvements in mobility, health outcomes, and overall quality of life through unstructured, daily-life activities such as walking, household chores, and recreational pursuits [9,10]. To amplify these health benefits, targeted nutritional supplementation has been increasingly proposed as a complementary strategy to address specific physiological deficits associated with aging. For instance, zinc supplementation has been shown to improve immune function, reduce low-grade chronic inflammation, and attenuate immunosenescence by restoring T-cell function in older adults [61,62]. Similarly, creatine monohydrate supplementation has demonstrated favorable effects on vascular endothelial function and metabolic profiles [63], while increased magnesium intake has been positively associated with improvements in bone mineral density in elderly populations [64]. Although these targeted nutritional interventions individually address specific aspects of age-related health decline, there remains limited evidence on the efficacy of dietary nucleotide supplementation in older adults who primarily engage in spontaneous physical activities. In contrast, studies conducted in younger or clinically defined populations engaged in structured exercise programs have consistently demonstrated that nucleotide supplementation provides ergogenic and immunomodulatory benefits, including enhanced adenosine triphosphate (ATP) regeneration, reduced oxidative stress, and improved inflammatory profiles [25,34,35,36,37]. These findings will be critically compared and discussed in this section alongside the results obtained in the present study.

### 4.2. Neurogenesis and Oxidative Stress Parameters

The experimental groups demonstrated substantial improvements in neurogenesis and oxidative stress parameters relative to the placebo. In terms of neurotrophic support, both nucleotide-supplemented groups demonstrated robust increases in BDNF levels (19.72% in the NF group and 10.13% in the YN group), while the placebo group showed only a marginal increase of 0.77%. Although there were no statistically significant differences between the two experimental groups, comparisons of each supplemented group with placebo confirmed significant effects, emphasizing the efficacy of nucleotide supplementation in enhancing neurogenesis. The pronounced increase in BDNF levels, particularly evident in the NF group, suggests enhanced neurogenesis and potential improvements in cognitive resilience among other adults. These results, coupled with the robust main effect of time (ES ≥ 0.91), demonstrate that dietary nucleotide supplementation effectively modulates neurotrophic support, a benefit absent in the placebo group. The NF supplement, composed solely of monophosphate nucleotides, exhibited a favorable trend toward greater BDNF levels, potentially due to the role of nucleotides in neurogenesis and neurotrophic support [65]. These specific blend of monophosphate nucleotides 5′ serve as precursors for nucleic acid synthesis, contributing to enhanced neuronal function, synaptic plasticity, and cellular repair mechanisms [66]. These neurotrophic benefits may also be mediated by signaling pathways related to increased substrate availability for neurogenesis and repair. Monophosphate nucleotides may promote neuronal differentiation and the upregulation of neurotrophic factors through enhanced DNA and RNA synthesis, as well as by facilitating neurotransmission and synaptic remodeling [66]. In this regard, the higher BDNF levels observed in the NF group suggest a mechanism of action aligned with these molecular processes, which may underlie the observed improvements in executive function. However, these differential effects did not reach statistical significance within the 10-week intervention period, suggesting that a longer duration may be necessary to detect meaningful distinctions between formulations.

Regarding oxidative stress, nucleotide supplementation resulted in significant improvements, as both experimental groups exhibited substantial reductions in F_2_-isoprostanes of 37.74% for NF and 26.44% for YN, in contrast to a minimal decrease of 3.54% in the placebo group. Similarly, the experimental groups experienced marked elevations in glutathione, with increases of 17.31% in NF and 14.88% in YN, whereas the placebo group demonstrated only a negligible increase of 1.24%. These differences reinforce the efficacy of nucleotide supplementation in attenuating oxidative damage, indicating an improved cellular redox environment; however, no significant differences were observed between the two supplemented groups, partially supporting our initial hypothesis.

In humans, direct evidence concerning the effects of nucleotide supplementation on specific oxidative stress biomarkers and neurotrophic factors remains limited. However, several indirect mechanisms have been proposed to explain potential antioxidant effects. These mechanisms include improved mitochondrial efficiency, leading to reduced reactive oxygen species (ROS) generation, as well as lower cortisol concentrations post-exercise [35,36], thereby decreasing cortisol-mediated oxidative damage. Our study provides the first direct evidence supporting these antioxidant effects in older adults who primarily engage in spontaneous physical activities, highlighting significant reductions in oxidative stress biomarkers and enhancements in neurotrophic support following nucleotide supplementation. Specifically, glutathione was positively correlated with BDNF (*ρ* = 0.08), suggesting that an enhanced antioxidant capacity may promote neurotrophic support and thereby bolster neuroprotection. Conversely, glutathione was negatively correlated with F_2_-isoprostanes (*ρ* = –0.16), indicating that higher antioxidant levels are associated with reduced oxidative damage. Collectively, these physiological mechanisms provide a plausible explanation for nucleotide-induced neuroprotective and muscle-preserving benefits, underscoring its potential role as an effective nutritional strategy to counteract oxidative stress in aging populations.

### 4.3. Inflammatory Parameters

Both IL-6 and TNF-α levels were significantly reduced in the nucleotide-supplemented groups compared to the placebo (*p* ≤ 0.001). Notably, the YN group exhibited the largest reduction (IL-6: −9.82%, TNF-α: −13.93%), followed by the NF group (IL-6: −3.64%, TNF-α: −8.57%), while the placebo group showed non-significant changes (IL-6: 7.83%, TNF-α: −3.17%). These reductions in pro-inflammatory cytokines indicate that dietary nucleotides can effectively modulate the inflammatory milieu, a crucial factor in counteracting the chronic, low-grade inflammation often associated with aging [67], with YN supplementation leading to a more pronounced reduction in both IL-6 and TNF-α levels. The more substantial anti-inflammatory response observed in the YN group may be attributed to its composition. Specifically, YN contains at least 40% total nucleotides, along with nucleosides and other bioactive compounds, including amino acids, oligonucleotides, minerals, group-B vitamins and peptides, which may enhance immune modulation [68]. These components play key roles in cell proliferation, tissue repair, and immune regulation, potentially contributing to more effective suppression of pro-inflammatory cytokines. This broader composition could influence immune cell signaling pathways, modulate inflammatory cascades, and reduce chronic low-grade inflammation [68]. Beyond these compositional considerations, the synergistic action of yeast-derived cofactors may also activate regulatory mechanisms such as antioxidant enzyme expression, and cytokine signaling modulation [22]. These effects could explain the greater reductions in systemic inflammatory markers observed in the YN group.

Moreover, our findings closely align with prior human research that has highlighted the immunomodulatory effects of nucleotide supplementation, including improved inflammatory profiles. Previous studies have documented elevated immunoglobulin A (IgA) activity following supplementation [34,35,36], reduced cortisol [35,36], and gastroprotective effects against irritable bowel-induced mucosal damage [25], collectively suggesting potential anti-inflammatory benefits. Notably, our study is the first to directly demonstrate significant reductions in systemic inflammatory biomarkers, specifically IL-6 and TNF-α, in older adults engaged in spontaneous physical activity, and potentially contribute to improved cognitive resilience, considering the well-established link between systemic inflammation and neurodegenerative processes [69]. This interpretation is further supported by the results of our correlation analysis, which revealed a positive relationship between IL-6 and TNF-α (*ρ* = 0.40), and a negative correlation between IL-6 and glutathione (*ρ* = −0.35), indicating that higher antioxidant levels correspond to reduced systemic inflammation and reinforcing the interconnected roles of IL-6 and TNF-α as pro-inflammatory cytokines. Therefore, our findings provide novel biochemical insights and highlight dietary nucleotide supplementation as a promising adjunct strategy for mitigating inflammation-related declines in aging populations.

### 4.4. Functional Capacities and Isokinetic Leg and Arm Strength

Regarding functional performance, in the 30 s Chair Stand Test, both NF and YN groups improved by more than 30%, while the placebo group improved by less than 10%. A similar pattern emerged in the Timed Up and Go Test, with the experimental groups demonstrating reductions of approximately 14–16% in completion times, whereas the placebo group showed only about a 2% improvement. Notably, a significant difference was found between the two nucleotide-supplemented groups (NF: –10.32%; YN: –5.85%; ES = 0.79), highlighting a nearly twofold greater improvement in mobility and functional agility induced by NF nucleotide supplementation. The 6 min Walk Test also reflected a significantly greater increase in distance covered for participants receiving nucleotides, surpassing the marginal gains observed in the placebo group. Previous research involving older adults engaging in unstructured physical activities has reported improvements in mobility and cardiorespiratory parameters, associating these types of low-intensity spontaneous activities with enhanced overall health [9,10]. In contrast, our data suggest that dietary nucleotides can amplify these benefits, resulting in more-pronounced enhancements in lower-limb functional performance, with greater improvements observed in the NF group compared to the YN group across all measured tests. However, the difference was statistically significant only in the Timed Up and Go Test, suggesting that the more neuro-targeted may be more effective in enhancing neuromuscular function.

Additionally, the present study demonstrated a significant main effect of time across all groups for isokinetic strength parameters, indicating that participants improved in all exercises measured. However, when comparing between groups, our analyses revealed notable differences in specific measures. In particular, for knee extension at 60°/s, the NF group exhibited significantly higher gains relative to the placebo group. Similarly, in elbow extension at 60°/s, both experimental groups showed significantly greater improvements compared to the placebo group. Although percentage improvements in knee and elbow flexion were higher in the experimental groups (with approximately 13.77% and 11.29% for knee flexion, and 4.29% and 13.11% for elbow flexion, respectively) compared to 6.27% and 2.16% in the placebo, these differences did not reach statistical significance. In this case, the trend in isokinetic strength improvements varied across all assessed variables. Further research is required to determine whether both formulations elicit differential effects on isokinetic strength, or whether they are similarly effective in supporting neuromuscular adaptations in older adults.

Regarding the bivariate correlation analysis, a significant positive association was observed between knee extension strength at 60%/s and muscle mass (*ρ* = 0.49), underscoring the critical role of preserving muscle mass for maintaining physical functionality. This result expands upon previous research, which reported trivial to moderate correlations and predictive capability between isokinetic knee and hip strength and functional performance measures, such as the 30 s Chair Stand Test, the 6 min walk test, and Timed Up and Go Test in older adults [70]. Furthermore, the observed negative correlations between handgrip strength and inflammatory (IL-6, *ρ* = −0.37; TNF-α, *ρ* = −0.24), as well as oxidative stress biomarkers (F_2_-isoprostanes, *ρ* = −0.30) underline the detrimental impact of systemic inflammation and oxidative damage on neuromuscular function. Collectively, these findings highlight the potential of nucleotide supplementation as a practical, non-pharmacological approach to boost functional independence and quality of life in aging populations.

### 4.5. Body Composition

In the nucleotide-supplemented groups, body fat mass, fat percentage, and muscle mass remained stable throughout the 10-week protocol (*p* ≥ 0.443), whereas the placebo group experienced a significant increase in body fat (5.04%) alongside a decline in muscle mass (−2.18%). These findings support our hypothesis, although no statistically significant differences were observed between the two nucleotide-supplemented groups. The correlation analyses further revealed a positive relationship between fat mass, body fat percentage, and IL-6 (*ρ* = 0.42 and *ρ* = 0.45, respectively), suggesting that greater adiposity may contribute to low-grade systemic inflammation. Conversely, muscle mass was negative correlated with IL-6 (*ρ* = −0.33) and positive associated with knee extension strength at 60°/s (*ρ* = 0.49), highlighting the protective role of preserving muscle mass to mitigate inflammation and support physical function. Additionally, the positive correlation between BDNF and muscle mass (*ρ* = 0.26) reinforces the proposed link between neurotrophic factors and neuromuscular adaptation. Collectively, our results suggest an amplifying effect of dietary nucleotide supplementation on body fat and muscle mass maintenance, by counteracting age-related increases in adiposity and the progressive muscle loss characteristic of sarcopenia.

### 4.6. Cognitive Functioning

Cognitive outcomes, assessed using the TMT, indicate that nucleotide supplementation exerts beneficial effects on executive function and related cognitive domains. Specifically, both nucleotide-supplemented groups demonstrated significant improvements in TMT-B performance compared to the placebo group (YN: −19.35%; NF: −18.21%; placebo: −4.31%), reflecting enhanced executive functioning, processing speed, mental flexibility, visual scanning, and search abilities. Regarding TMT-A, percent improvements from baseline were slightly greater in the nucleotide-supplemented groups (YN: −20.40%; NF: −20.24%), than in the placebo group (−9.20%). Although between-group differences for TMT-A were not statistically significant, both experimental groups showed significant within-group improvements, suggesting a beneficial effect of dietary nucleotides on cognitive performance. Furthermore, TMT-B performance showed a positive correlation with BDNF levels (*ρ* = 0.26), reinforcing the concept that higher neurotrophic support is associated with enhanced cognitive flexibility, executive function, and processing speed. These findings collectively suggest that dietary nucleotide supplementation over a 10-week period effectively supports cognitive resilience, particularly in executive functioning domains, in older adults engaged in spontaneous physical activities. Given the current absence of studies directly assessing cognitive function following nucleotide supplementation in physically independent older adults, our findings can be contextualized through comparison with the preclinical research of Chen et al. [31]. These authors investigated the effects of nucleoside and nucleotide supplementation in senescence-accelerated mice over a 14-week intervention period, demonstrating significant improvements in memory performance on passive and active avoidance tests among aged mice. Additionally, supplemented animals exhibited reduced brain lipofuscin accumulation and fewer cerebral vacuoles, indicative of attenuated neurodegenerative damage. Therefore, although our results support the hypothesis that nucleotides possess significant anti-aging properties with the potential to attenuate age-related cognitive decline, further research employing similar protocols in older adults is imperative to establish robust evidence.

### 4.7. Physical and Mental Health-Related Quality of Life Dimensions

All three conditions showed significant improvements in physical and mental health-related quality of life. However, both nucleotide-supplemented groups experienced significantly greater enhancements compared to the placebo group, particularly in the dimensions of general health, bodily pain, vitality, and mental health, with moderate to large effect sizes (ES ranging from 0.62 to 1.19). A plausible explanation for the improvements observed across all three groups in our study may be related to the psychological and mental health benefits commonly associated with unstructured, low-intensity activities, such as walking at a pace slower than 100 steps per minute, which has been significantly associated with fewer depressive symptoms, and enhanced quality of life [10]. Nevertheless, our findings extend these observations by demonstrating that dietary nucleotide supplementation can further amplify the psychological and functional health benefits derived from spontaneous physical activity.

The correlation analysis revealed significant associations between health-related quality of life dimensions and several physical, cognitive, and physiological parameters. Vitality exhibited moderate positive correlations with functional performance measures such as the 30 s Chair Stand Test (*ρ* = 0.33), manual grip strength (*ρ* = 0.27), and isokinetic knee extension strength at 60°/s (*ρ* = 0.31), highlighting the role of improved muscular capacity in enhancing perceived energy and vigor. Similarly, vitality positively correlated with mental health (*ρ* = 0.30), and general health (*ρ* = 0.44), indicating it integral contribution to overall well-being. Furthermore, bodily pain was inversely associated with both physical function tests (*ρ* ranging from −0.19 to −0.24) and muscular strength, particularly isokinetic elbow extension strength at 60°/s (*ρ* = −0.21), suggesting that reduced pain perception might contribute to improved functional outcomes. General health exhibited positive correlations with lower limb functional performance, including the 30 s Chair Stand Test (*ρ* = 0.38) and 6 min Walk Test (*ρ* = 0.29), underlining the significance of physical function in perceived overall well-being. Mental health scores also showed positive associations with general health (*ρ* = 0.44) and negative correlations with bodily pain (*ρ* = −0.29), underscoring the interplay between psychological wellness and pain perception in older adults. Collectively, these correlations indicate that improvements in muscular strength and physical function observed with dietary nucleotide supplementation may contribute to enhanced health-related quality of life, demonstrating the potential benefits of this nutritional strategy in physically independent older adults.

### 4.8. Limitations and Future Research

Although the present study employed a rigorously designed randomized controlled trial, several limitations should be acknowledged. Firstly, the study sample exclusively comprises physically independent older adults who regularly engage in spontaneous, low-intensity physical activities. This specific selection criteria limits the generalizability of our findings to older populations characterized by sedentary lifestyles or those presenting clinical conditions. Moreover, subsequent studies should encompass larger, more diverse populations, including individuals with varying physical activity profiles, functional statuses, and underlying medical conditions, thereby improving external validity. Secondly, the 10-week intervention duration may have been insufficient to fully capture some long-term physiological adaptations. Extending the intervention period could potentially reveal more information about the differences between the two nucleotides formulations. Thirdly, while our measurements of key physiological biomarkers provided valuable peripheral insights, these serum markers may not directly correspond to central nervous system alterations. Additionally, examining detailed molecular pathways, specifically those involved in mitochondrial function, ATP regeneration, and inflammatory regulation, may help elucidate the precise mechanisms through which nucleotides exert their protective effects. Finally, investigating various supplementation dosages represents an important future research avenue, which may help determine the optimal nucleotide intake necessary for maximizing functional, cognitive, and metabolic benefits in older adults.

## 5. Conclusions

The present study demonstrated that dietary nucleotide supplementation combined with spontaneous physical activity significantly improved physical function, cognitive performance, metabolic health, oxidative stress biomarkers, and inflammatory profiles in physically independent older adults. Both nucleotide formulations effectively mitigated age-related adiposity gains and muscle loss, suggesting a protective role against sarcopenia, though no significant differences emerged between formulations within 10 weeks. However, the YN group showed a trend toward greater inflammatory modulation, while the NF group exhibited more pronounced neurotrophic benefits and superior functional improvements, as evidenced by its significantly greater reduction in Timed Up and Go Test time, underscoring its potential advantage in enhancing neuromuscular function and mobility. Robust reductions in inflammatory cytokines and oxidative damage further support nucleotides as a valuable nutritional strategy for promoting healthy aging and potentially informing future public health initiatives. Future studies should clarify optimal dosage and long-term effects in diverse older populations.

## Figures and Tables

**Table 1:** Intervention effects on the brain-derived neurotrophic factor and oxidative stress parameters.

Variable	Group	Mean ± SD (Pre-Test)	Mean ± SD (Post-Test)	Δ%	*p*-Value (Time)	ES (Time)	Comparison (Group)	*p*-Value (Group)	ES (Group)
*Brain-derived neurotrophic factor* (pg/mL)	(1) YN	43,057.43 ± 5442.02	48,387.72 ± 5882.78	12.38	**0.004**	0.91	2–3	0.048	0.53
	(2) NF	37,586.04 ± 5892.84	44,367.36 ± 6098.58	18.04	**0.008**	1.09			
	(3) Placebo	34,437.03 ± 5823.99	35,494.92 ± 5964.15	3.07	0.339	0.18			
*F_2_-Isoprostanes* (pg/mL)	(1) YN	3378.30 ± 674.28	2028.40 ± 750.70	−39.96	**<0.001**	−1.82	1–3	0.031	−0.88
	(2) NF	3805.36 ± 692.94	1732.43 ± 657.76	−54.47	**<0.001**	−2.97	2–3	0.014	−1.50
	(3) Placebo	2804.37 ± 608.72	2407.54 ± 657.49	−14.15	0.103	−0.60			
*Glutathione* (µIU/mL)	(1) YN	205.76 ± 34.98	323.07 ± 28.30	12.78	**0.007**	0.80	1–3	0.034	0.19
	(2) NF	204.64 ± 36.08	229.20 ± 30.07	12.00	**0.012**	0.72	2–3	0.046	0.22
	(3) Placebo	212.93 ± 42.15	219.64 ± 34.89	3.15	0.068	0.17			

Data are presented as means ± standard deviations. Significant pairwise time differences are highlighted in bold. %Δ represents the percent change between pre- and post-intervention, calculated as [(post-test score − pre-test score)/pre-test score] × 100. YN, yeast nucleotides formulation; NF, neuro-based nucleotide formulation; ES, Hedge’s g effect size.

**Table 2:** Intervention effects on the inflammatory parameters.

Variable	Group	Mean ± SD (Pre-Test)	Mean ± SD (Post-Test)	Δ%	*p*-Value (Time)	ES (Time)	Comparison (Group)	*p*-Value (Group)	ES (Group)
*Interleukin-6* (pg/mL)	(1) YN	12.83 ± 2.26	11.57 ± 1.54	−9.82	**0.001**	−0.63	1–3	<0.001	−0.23
	(2) NF	12.07 ± 2.50	11.63 ± 2.47	−3.64	**<0.001**	−0.18	2–3	0.013	−0.52
	(3) Placebo	13.40 ± 2.51	14.45 ± 2.98	7.83	0.181	0.37			
*Tumor necrosis factor alpha* (pg/mL)	(1) YN	32.43 ± 5.40	27.91 ± 4.82	−13.93	**<0.001**	−0.85	1–3	0.006	−0.17
	(2) NF	31.48 ± 4.52	28.78 ± 4.36	−8.57	**<0.001**	−0.59	2–3	0.023	−0.09
	(3) Placebo	31.51 ± 5.41	30.51 ± 4.13	−3.17	0.453	−0.20			

Data are presented as means ± standard deviations. Significant pairwise time differences are highlighted in bold. %Δ represents the percent change between pre- and post-intervention, calculated as [(post-test score − pre-test score)/pre-test score] × 100. YN, yeast nucleotide formulation; NF, neuro-based nucleotide formulation; ES, Hedge’s g effect size.

**Table 3:** Intervention effects on the functional capacities.

Variable	Group	Mean ± SD (Pre-Test)	Mean ± SD (Post-Test)	Δ%	*p*-Value (Time)	ES (Time)	Comparison (Group)	*p*-Value (Group)	ES (Group)
*30 s chair stand* (repetitions)	(1) YN	15.56 ±3.36	17.04 ± 4.59	9.51	0.080	0.36	1–3	0.006	0.77
	(2) NF	14.88 ± 2.50	17.64 ± 3.72	18.54	**<0.001**	0.84	2–3	<0.001	1.05
	(3) Placebo	14.52 ± 3.02	13.95 ± 3.18	−3.92	**0.001**	−0.18			
*Timed-up and go test* (seconds)	(1) YN	5.63 ± 0.61	5.30 ± 0.53	−5.86	**0.004**	−0.56	1–2	0.014	0.79
	(2) NF	5.4 2± 0.64	4.86 ± 0.57	−10.32	**<0.001**	−0.90	1–3	0.018	−0.78
	(3) Placebo	5.70 ± 0.56	5.74 ± 0.58	0.70	**0.008**	0.07	2–3	<0.001	−1.50
*6 min walk test* (meters)	(1) YN	576.02 ± 63.32	565.08 ± 61.51	−1.90	0.181	−0.17	1–2	<0.001	−1.10
	(2) NF	616.62 ± 62.07	634.88 ± 62.52	2.96	**0.021**	0.28	2–3	0.014	0.62
	(3) Placebo	605.92 ± 51.24	598.90 ± 50.90	−1.15	0.409	−0.13			
*Maximal grip strength* (kilograms)	(1) YN	26.26 ± 7.17	26.54 ± 7.85	1.06	0.650	0.04	1–2	0.045	−0.79
	(2) NF	32.56 ± 9.53	34.14 ± 10.89	4.85	**0.015**	0.15	2–3	0.038	0.14
	(3) Placebo	32.85 ± 10.16	32.64 ± 9.81	−0.64	0.467	−0.04			

Data are presented as means ± standard deviations. Significant pairwise time differences are highlighted in bold. %Δ represents the percent change between pre- and post-intervention, calculated as [(post-test score − pre-test score)/pre-test score] × 100. YN, yeast nucleotide formulation; NF, neuro-based nucleotide formulation; ES, Hedge’s g effect size.

**Table 4:** Intervention effects on the isokinetic leg and arm strength.

Variable	Group	Mean ± SD (Pre-Test)	Mean ± SD (Post-Test)	Δ%	*p*-Value (Time)	ES (Time)	Comparison (Group)	*p*-Value (Group)	ES (Group)
*Knee flexion 60*°/*s* (N·m)	(1) YN	43.26 ± 13.82	49.22 ± 15.27	13.77	**<0.001**	0.40			
	(2) NF	53.84 ± 18.24	59.92 ± 16.81	11.29	**0.009**	0.34			
	(3) Placebo	58.47 ± 18.37	62.14 ± 19.46	6.27	**<0.001**	0.19			
*Knee extension 60*°/*s* (N·m)	(1) YN	81.39 ± 21.40	94.63 ± 21.51	16.26	**<0.001**	0.60	2–3	0.009	0.19
	(2) NF	96.72 ± 34.60	116.12 ± 33.05	20.05	**<0.001**	0.56			
	(3) Placebo	101.38 ± 34.04	109.47 ± 36.86	7.97	**<0.001**	0.23			
*Elbow flexion 60*°/*s* (N·m)	(1) YN	17.47 ± 9.28	18.22 ± 6.57	4.29	**0.046**	0.10			
	(2) NF	22.88 ± 11.11	25.88 ± 11.86	13.11	**0.001**	0.25			
	(3) Placebo	24.47 ± 12.80	25.00 ± 12.91	2.16	**<0.001**	0.04			
*Elbow extension 60*°/*s* (N·m)	(1) YN	35.00 ± 8.75	40.18 ± 9.62	14.79	**<0.001**	0.54	1–3	0.022	−0.64
	(2) NF	42.80 ± 12.70	51.36 ± 14.70	20.00	**<0.001**	0.60	2–3	0.000	0.014
	(3) Placebo	45.90 ± 16.00	49.23 ± 17.14	7.25	**<0.001**	0.19			

Data are presented as means ± standard deviations. Significant pairwise time differences are highlighted in bold. %Δ represents the percent change between pre- and post-intervention, calculated as [(post-test score − pre-test score)/pre-test score] × 100. YN, yeast nucleotide formulation; NF, neuro-based nucleotide formulation; ES, Hedge’s g effect size.

**Table 5:** Intervention effects on the body composition.

Variable	Group	Mean ± SD (Pre-Test)	Mean ± SD (Post-Test)	Δ%	*p*-Value (Time)	ES (Time)	Comparison (Group)	*p*-Value (Group)	ES (Group)
*Body fat mass* (kilograms)	(1) YN	24.24 ± 6.85	24.38 ± 6.53	0.58	0.911	0.03	1–3	<0.001	−1.24
	(2) NF	23.59 ± 5.42	23.53 ± 4.99	−0.25	0.913	−0.02	2–3	<0.001	−1.53
	(3) Placebo	29.29 ± 6.11	32.89 ± 6.88	12.39	**<0.001**	0.53			
*Body fat percentage* (%)	(1) YN	36.83 ± 7.14	36.86 ± 6.80	0.08	0.614	0.01	1–3	0.005	−0.41
	(2) NF	33.94 ± 6.34	33.96 ± 5.95	0.05	0.824	0.01	2–3	<0.001	−0.87
	(3) Placebo	37.83 ± 6.25	39.74 ± 7.08	5.04	**<0.001**	0.28			
*Body muscle mass* (kilograms)	(1) YN	39.51 ± 8.31	39.45 ± 7.90	−0.15	0.670	−0.01			
	(2) NF	44.32 ± 10.22	44.24 ± 10.19	−0.18	0.443	−0.01			
	(3) Placebo	46.78 ± 11.57	45.76 ± 11.32	−2.18	**<0.001**	−0.09			

Data are presented as means ± standard deviations. Significant pairwise time differences are highlighted in bold. %Δ represents the percent change between pre- and post-intervention, calculated as [(post-test score − pre-test score)/pre-test score] × 100. YN, yeast nucleotide formulation; NF, neuro-based nucleotide formulation; ES, Hedge’s g effect size.

**Table 6:** Intervention effects on cognitive functioning.

Variable	Group	Mean ± SD (Pre-Test)	Mean ± SD (Post-Test)	Δ%	*p*-Value (Time)	ES (Time)	Comparison (Group)	*p*-Value (Group)	ES (Group)
*TMT-A* (seconds)	(1) YN	45.39 ± 13.90	36.13 ± 10.98	−20.40	**<0.001**	−0.71			
	(2) NF	42.88 ± 10.26	34.20 ± 7.04	−20.24	**<0.001**	−0.96			
	(3) Placebo	41.38 ± 12.63	37.57 ± 6.77	−9.20	0.076	−0.36			
*TMT-B* (seconds)	(1) YN	111.65 ± 49.19	90.04 ± 38.45	−19.35	**<0.001**	−0.47	1–3	0.012	−0.04
	(2) NF	86.28 ± 30.02	70.56 ± 18.40	−18.21	**<0.001**	−0.61	2–3	<0.001	−0.92
	(3) Placebo	94.71 ± 27.66	90.38 ± 23.54	−4.31	0.286	−0.16			

Data are presented as means ± standard deviations. Significant pairwise time differences are highlighted in bold. %Δ represents the percent change between pre- and post-intervention, calculated as [(post-test score − pre-test score)/pre-test score] × 100. YN, yeast nucleotide formulation; NF, neuro-based nucleotide formulation; ES, Hedge’s g effect size.

**Table 7:** Intervention effects on the physical and mental health-related quality of life dimensions.

Variable	Group	Mean ± SD (Pre-Test)	Mean ± SD (Post-Test)	Δ%	*p*-Value (Time)	ES (Time)	Comparison (Group)	*p*-Value (Group)	ES (Group)
*General health* (score)	(1) YN	71.73 ± 12.57	88.26 ± 8.34	23.04	**<0.001**	1.49	1–3	0.047	0.62
	(2) NF	70.00 ± 11.98	91.40 ± 6.21	30.57	**<0.001**	2.17	2–3	0.002	1.05
	(3) Placebo	72.61 ± 16.85	82.38 ± 10.20	13.45	**0.002**	0.68			
*Mental health* (score)	(1) YN	80.34 ± 12.98	91.65 ± 8.60	14.07	**<0.001**	0.99	1–3	0.004	0.87
	(2) NF	80.64 ± 10.04	91.36 ± 6.07	13.29	**<0.001**	1.25	2–3	0.003	0.96
	(3) Placebo	79.00 ± 14.42	83.76 ± 9.12	6.02	**0.022**	0.38			
*Vitality* (score)	(1) YN	68.69 ± 17.59	88.13 ± 7.26	28.30	**<0.001**	1.40	1–3	0.009	0.83
	(2) NF	70.20 ± 12.86	86.76 ± 7.28	23.58	**<0.001**	1.54	2–3	0.023	0.67
	(3) Placebo	68.80 ± 18.43	80.95 ± 9.69	17.65	**0.001**	0.80			
*Bodily pain* (score)	(1) YN	82.60 ± 11.52	95.65 ± 5.06	15.79	**<0.001**	1.41	1–3	<0.001	1.19
	(2) NF	80.84 ± 15.68	93.60 ± 6.37	15.78	**<0.001**	1.03	2–3	0.003	0.89
	(3) Placebo	78.38 ± 16.61	85.90 ± 10.14	9.59	**0.005**	0.53			

Data are presented as means ± standard deviations. Significant pairwise time differences are highlighted in bold. %Δ represents the percent change between pre- and post-intervention, calculated as [(post-test score − pre-test score)/pre-test score] × 100. YN, yeast nucleotide formulation; NF, neuro-based nucleotide formulation; ES, Hedge’s g effect size.

## Data Availability

The database of the present study can be downloaded through the following link: https://osf.io/d2kjg/ (accessed on 8 April 2025).
